# Does Hospital Competition Harm Inpatient Quality? Empirical Evidence from Shanxi, China

**DOI:** 10.3390/ijerph15102283

**Published:** 2018-10-17

**Authors:** Xiaojun Lin, Miao Cai, Qiang Fu, Kevin He, Tianyu Jiang, Wei Lu, Ziling Ni, Hongbing Tao

**Affiliations:** 1 Department of Health Administration, School of Medicine and Health Management, Tongji Medical College, Huazhong University of Science and Technology, Wuhan 430030, China; xjlin@hust.edu.cn (X.L.); jtychris@163.com (T.J.); tongjiweilu@hust.edu.cn (W.L.); zilingziling@hust.edu.cn (Z.N.); 2 Department of Epidemiology and Biostatistics, College for Public Health and Social Justice, Saint Louis University, St Louis, MO 63104, USA; miao.cai@slu.edu (M.C.); john.fu@slu.edu (Q.F.); 3 Department of Biostatistics, School of Public Health, University of Michigan, Ann Arbor, MI 48109, USA; kevinhe@umich.edu

**Keywords:** Hospital competition, acute myocardial infarction, inpatient quality of care, in-hospital mortality

## Abstract

This study aimed to determine whether hospital competition is associated with improved in-hospital mortality in Shanxi, China. We included a total of 46,959 hospitalizations for acute myocardial infarction (AMI) and 44,063 hospitalizations for pneumonia from 2015 to 2017. Hospital competition was measured as Herfindahl–Hirschman Index based on the patient predicted flow approach. Two-level random-intercept logistic models were applied to explore the effects of hospital competition on quality for both AMI and pneumonia diagnoses. Hospital competition exerts negative or negligible effects on inpatient quality of care, and the pattern of competition effects on quality varies by specific diseases. While hospital competition is insignificantly correlated with lower AMI in-hospital mortality (odds ratio (OR): 0.94, 95% confidence interval (CI): 0.77–1.11), high hospital competition was, in fact, associated with higher in-hospital mortality for pneumonia patients (OR: 1.99, 95% CI: 1.51–2.64). Our study suggests that simply encouraging hospital competition may not provide effective channels to improve inpatient quality of health care in China’s current health care system.

## 1. Introduction

Since the new round of healthcare reforms implemented in April 2009, China has made great achievements in widening the availability of health care services, i.e., expanding health insurance coverage and establishing national programs for essential medicine [1,2]. However, coinciding with the rising incomes and increasing health insurance coverage in China, people’s demand for health services and expectations for quality care are growing rapidly [2,3]. How to improve efficiency and quality of the health care system has become daunting challenges for deepening the healthcare reforms. In China, hospitals are classified into three levels: Primary (level 1), secondary (level 2), and tertiary (level 3) through the hospital accreditation system [4]. The accreditation is conducted every four years by the accreditation agency, and the major classification criteria are the size of hospitals (e.g., beds) and the number and type of medical equipment (e.g., magnetic resonance imaging). Hospitals in lower levels have strong incentives to be promoted to higher levels, and hospitals in high levels tend to maintain their existing accreditation status. As a result, hospitals have incentives to expand bed size, introduce high-technology therapies and purchase new medical equipment, which is known as the “medical arms race” [5].

Similar to more developed countries such as the United States and United Kingdom, the Chinese government also emphasizes the role of market competition in its healthcare reforms and induces both external and internal competition into its health industry [6]. The external competition is between public and private hospitals. While public hospitals still dominate China’s healthcare market in terms of health care delivery and medical resources, the competition between public and private hospitals is expected to become more intense due to the recent policy of encouraging private capital to improve the healthcare system [7,8]. The internal competition occurs among individual hospitals, where they compete on service quantity, quality of care and cost per patient, regardless of the ownership and hospital accreditation level [5]. While the accreditation system is designed to guide patient referrals, the referral system that has been established in China is not effective. Patients are free to seek care in any provider they prefer and can afford [9]. Usually, patients prefer secondary and tertiary hospitals that provide comprehensive care as well as medical education and research and believe that these hospitals provide high-quality services [6,10]. The operation objective of the private hospitals is to make profits, so they have incentives to attract patients. Public hospitals are managed and subsidized by the government and have the characteristic of public welfare. However, the subsidies from government in public hospitals have been declined since the fiscal system reforms of the 1980s [11]. Currently, most public hospitals are often financially self-reliant, and the hospital gross income is financed largely from operating income, such as fees for prescription drugs and diagnostic services, rather than government subsidies [12]. Therefore, in the context of hospital competition in China, both public and private hospitals are incentivized to increase the number of patients. Additionally, the public hospitals and most of the private hospitals accept the medical pricing regulations from the government [6].

The effect of market competition on health quality has received considerable attention in recent years. In classic economic theories, increased competition leads to mobilizing productivity effectively and broadly in industries such as banking and computers [13]. Many countries have introduced market mechanisms and competition into their health industries, attempting to improve the overall health quality [14,15,16]. However, previous studies suggested that the effects of market competition on medical quality were controversial [17]. For example, using Herfindahl-Hirschman Index (HHI) as the measure of competition, Kessler et al. [18], Cooper et al. [19], and Gaynor et al. [20] found increasing hospital competition reduced the mortalities of acute myocardial infarction (AMI) patients. Bloom et al. [21] extended the measures of hospital quality and found higher hospital competition was significantly associated with the better management quality for AMI patients. Using the data from NHS, Propper et al. [22] studied the impact of competition on mortality and found that increases in hospital competition were associated with higher hospital death rates. Some scholars found mixed results in their studies. Using mortality as a measure of quality for AMI and pneumonia, Gowrisankaran and Town [23] reported that increasing competition for Health Maintenance Organization patients was directly correlated to a reduction of mortality for AMI, but increasing competition for Medicare patients was related to higher mortality for both diagnoses. Palangkaraya and Yong [24] found that hospitals in high competition markets had lower readmission rates but higher mortality rates. Mutter et al. [16] and Wong et al. [25] found that the effects varied by the measures of quality and competition. Other researchers found no statistically significant relationship between competition and quality in any area [26,27].

Despite the abundant literature exploring the effects of competition on quality, the studies mainly focused on health care systems of developed countries such as the US (majority) and the UK, while evidence from developing countries remains scant. Recently, Pan et al. [6] analyzed the impacts of hospital competition on quality and cost in China, using both provincial and individual-level data, and found that higher hospital competition could improve the performance of health care delivery. Yang and Pan [28] reported an inversed U-shaped relationship between HHI and medical disputes. However, data used in these studies did not include comprehensive and detailed data on both hospitals and patients. Furthermore, endogeneity is the major concern in estimating the impact of hospital competition on quality of care, since high-quality hospitals may attract more patients and have more market power [25].

This paper aims to investigate the potential relationship between hospital competition and quality using administrative data of patients with AMI and pneumonia in Shanxi, China. Following Kessler and McClellan [18], a predicted patient flow is used to calculate the HHI to address the endogeneity problem. Previous studies from China suggested that competition can improve quality [6], therefore, in this paper, we hypothesized that more intense hospital competition is associated with better quality of care for patients with AMI and pneumonia.

## 2. Materials and Methods

### 2.1. Data Source

The Shanxi Province is in Northern China, with a total area of 156,700 square kilometers. There are 11 administrative divisions in Shanxi, and Taiyuan is the capital city. According to the latest Statistical Yearbook of Shanxi, there were 36.8 million residents in Shanxi, with 56.2% living in urban areas and 35,303 yuan of per capita gross domestic product in 2016 [29].

In this study, we used two data sources obtained from the Health and Family Planning Commission of Shanxi. The first one is the front pages of inpatients medical records derived from the Hospital Discharge Database operated by the Center for Health Statistics Information of Shanxi. The database covers all hospitals in Shanxi and contains over 200 variables including unique provider ID, patients’ demographic characteristics (e.g., age, gender, race/ethnicity, and insurance status), principal diagnosis (identified using the International Classification of Diseases, 10th Revision (ICD-10) codes), up to 10 secondary diagnoses (identified using ICD-10 codes), up to 7 procedures (identified using the International Classification of Diseases, Ninth Revision, Clinical Modification codes), length of stay, medical expenses in sub-categories, and outcomes (e.g., discharge status, hospital infection and adverse events during hospitalization). The second source is the summary statistics of health providers (hospitals and clinics) from 2015 to 2017, collected annually by the National Survey System of Health Resource and Medical Service. The summarized data include basic information (e.g., ownership, accreditation level and address), staff (e.g., doctors, nurses and pharmacists), bed size, building and infrastructure, medical equipment, income and expenditure, and medical service. We extracted the information of health providers from this database, including provider IDs, location, accreditation, ownership, beds, and staff. Using the unique provider IDs, we linked patients’ and providers’ information from these two data sources. All patient unique identifiers such as names and ID card numbers were excluded prior to the authors’ access.

### 2.2. Study Population

To examine the relationship between hospital competition and quality, we focused on AMI and pneumonia similar to Gowrisankaran and Town [23]. First, AMI and pneumonia are the leading causes for hospitalization and death in China. Second, AMI and pneumonia have been frequently used in previous studies from United States and United Kingdom, allowing us to compare our results with these studies. Third, the effect of hospital competition on quality of AMI and pneumonia patients may be different. For patients admitted with AMI, they may exercise less discretion in choosing hospitals since the time from symptom onset to hospital admission is particularly important to patients’ prognosis [30]. While hospitals may not compete directly for AMI patients, they do compete to maintain their hospital accreditation level. The management of AMI impacts the hospital accreditation results, which incentivizes hospitals to provide quality care for AMI. Pneumonia patients, however, have discretion in choosing the hospital for admission and travel time is less likely to impact their prognosis; thus, hospitals may compete directly for pneumonia patients [23].

**Figure 1:**
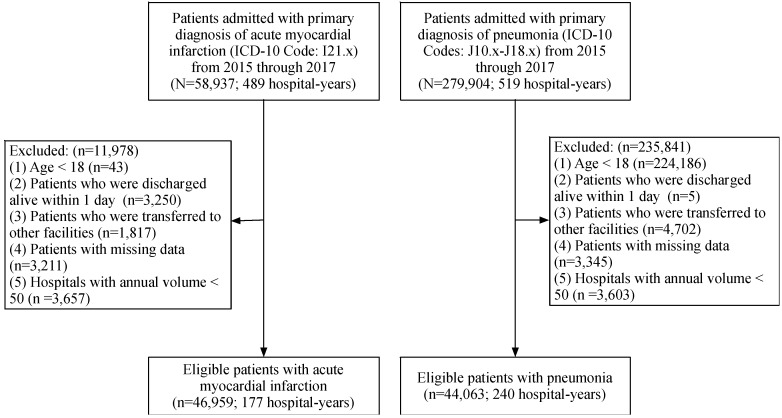
Flow diagram of the processing of patient-level data.

Using the ICD-10 of principal diagnosis with variations in the three digits after the decimal point, we extracted a sample of AMI (ICD-10: I21.x) and pneumonia (ICD-10 codes: J10.x-J18.x) patients who were over 18 years old and admitted between 2015 and 2017. The sampling process is displayed in Figure 1. We excluded patients who were discharged alive within 1 day after admission because they were likely to leave against medical advice and the treatment time was very limited. Patients with missing data such as age, gender, admission status, insurance, and discharge status were also excluded. In addition, we excluded patients who were transferred to another hospital or community health service center because their admissions were truncated and subsequent treatment information in other facilities was unavailable for our study. Furthermore, we dropped hospitals with fewer than 50 cases annually to prevent one death case from unduly affecting the hospital mortality rate, which would lead to measurement error in the quality measures. while some very small volume hospitals were excluded, the hospitals included represented most of the hospital care provided to a majority of residents in Shanxi. A total of 46,959 hospitalizations for AMI and 44,063 hospitalizations for pneumonia were included in our analyses.

### 2.3. Variables of Interest

In this paper, we focus on in-hospital mortality as the indicator of hospital quality, because it is the most common outcome-based measure used throughout the literature. In-hospital mortality was defined as all deaths that occurred during the hospitalization.

To avoid potential problems of endogeneity, we follow the predicted patient flow approach used in Kessler and McClellan [18], Gowrisankaran and Town [23], and Gaynor et al. [20]. As described by Kessler and McClellan [18], this approach defines a potential market that captures the potentially competitive hospitals, rather than defining geographic markets arbitrarily. It allows us to calculate the expected patient shares based on exogenous determinants of patient flows, rather than potentially endogenous measures (e.g., actual hospital of admission and bed capacity). Hospital competition was measured based on predicted patient flow using a conditional logit model of hospital choice. The model of hospital choice accounts for variables that are exogenous to unobserved characteristics of patients and hospitals. Specifically, we performed the following three steps to calculate the competition:

In the first step, we specify the following model of patients’ hospital choice, and assume that patients choose the hospital that maximizes their utility:

(1) $$U_{ij}=\sum_{h=1}^{3}\left\{\begin{array}{c} \theta_{1}^{h}\left(d_{ij}-d_{ij^{+}}^{h}\right)z_{j}^{h}+\theta_{2}^{h}\left(d_{ij}-d_{ij^{+}}^{h}\right)\left(1-z_{j}^{h}\right)\\ +\theta_{3}^{h}\left(d_{ij}-d_{ij^{-}}^{h}\right)z_{j}^{h}+\theta_{4}^{h}\left(d_{ij}-d_{ij^{-}}^{h}\right)\left(1-z_{j}^{h}\right)\\ +\theta_{5}^{h}\left(female_{i}\times z_{j}^{h}\right)\\ +\theta_{6}^{h}\left(young_{i}\times z_{j}^{h}\right)+\theta_{7}^{h}\left(old_{i}\times z_{j}^{h}\right)\\ +\theta_{8}^{h}\left(lowseverity_{i}\times z_{j}^{h}\right)+\theta_{9}^{h}\left(highseverity_{i}\times z_{j}^{h}\right)\\ +\theta_{10}^{h}\left(emergency_{i}\times z_{j}^{h}\right)\\ +\theta_{11}^{h}\left(Elixhauser_{i}\times z_{j}^{h}\right)+\varepsilon_{ij} \end{array}\right\},$$

where $U_{ij}$ denotes the utility patient i receives from hospital j∈J; patients’ choice sets J were restricted to their chosen hospital and all hospitals within 100 km fixed radius; $d_{ij}$ denotes the distance from the address of patient i to hospital j; $d_{ij^{+}}^{h}$ and $d_{ij^{-}}^{h}$ denote the distance to the closest hospital as a good or poor substitute for hospital j in terms of characteristic h, respectively; $z_{j}^{1}$ denotes whether hospital j is a tertiary hospital; $z_{j}^{2}$ denotes whether hospital j is a big hospital (defined as hospital beds over the median beds for a specific disease and year); $z_{j}^{3}$ denotes whether hospital j is a public hospital. Patients are divided into three categories by age: Young (18 to 40), mid (41 to 65), and old (over 65). $female_{i}$ denotes the gender of patient i and $emergency_{i}$ indicates whether patient i is admitted through the emergency department. Dummies are also included for low and high severity (respectively, any patient with only one diagnosis code, or with three or more diagnosis codes in their secondary diagnoses). Charlson Comorbidity Index and Elixhauser Comorbidity Index (ECI) are good measures of patient comorbidities, though the ECI performs slightly better in predicting patients’ mortality [31,32]. The ECI is a weighted score of 30 comorbidities, where a larger value indicates more severe comorbidities [33].

In the second step, we use the above model of hospital choice to predict the probability of patient i with disease s admitted to hospital j in their choice set J:

(2) $${\hat{P}}_{ij}^{s}=\frac{\exp\left({\hat{u}}_{ij}^{s}\right)}{\sum_{k\in J}\exp\left({\hat{u}}_{ik}^{s}\right)},$$

where ${\hat{u}}_{ij}^{s}$ is the expected mean utility of being admitted to hospital *j*. The HHI for patient i with disease s is:

(3) $$HHI_{i}^{s}=\sum_{k\in J}{\left({\hat{P}}_{ij}^{s}\right)}^{2},$$

In the third step, the calculation of HHI for hospital j for disease s is:

(4) $$HHI_{j}^{s}=\frac{1}{N_{j}^{s}}\sum_{i\in I^{s}}{\hat{P}}_{ij}^{s}HHI_{i}^{s},$$

where $I^{s}$ is the set of patients with disease s and $N_{j}^{s}=\sum_{i\in I^{s}}{\hat{P}}_{ij}^{s}$. The $N_{j}^{s}$ indicates the expected volume of hospital j for disease s. The $HHI_{j}^{s}$ takes on values between zero to one, where $HHI_{j}^{s}=1$ denotes that hospital *j* is a monopolist and $HHI_{j}^{s}=0$ means hospitals in the defined market compete perfectly. To make the interpretation of results more intuitive, we construct the negative natural logarithm of $HHI_{j}^{s}$ as follows:

(5) $$Comp_{j}^{s}=-ln\left(HHI_{j}^{s}\right)=ln\left(\frac{1}{HHI_{j}^{s}}\right),$$

where $Comp_{j}^{s}$ is the measure of market competition for hospital j and disease s, with zero corresponding to monopoly and infinity to perfect competition.

### 2.4. Statistical Analysis

Since patient admissions are nested within hospitals and the competition measure is invariant to admissions in a given hospital, we apply a two-level random-intercept logistic model to estimate the effect of hospital competition on quality and control potential sources of heterogeneity that may affect quality. Specifically, we use the general form of the model as follows:

(6) $$y_{ij}\sim \mathrm{Bern}\left(p_{ij}\right),$$

(7) $$\ln\left(\frac{p_{ij}}{1-p_{ij}}\right)=\beta_{0}+\beta_{1}X_{ij}+\beta_{2}Z_{j}+u_{j},$$

(8) $$u_{j}\sim N\left(0,\sigma_{u}^{2}\right),$$

where $y_{ij}$ is a binary variable with the value of one if patient i who was admitted to hospital j died during hospitalization; otherwise, $y_{ij}$ has a value of zero. $y_{ij}$ has an independent Bernoulli distribution with the parameter $p_{ij}$. $X_{ij}$ is a vector containing patient characteristics, and $Z_{j}$ represents the hospital characteristics and competition variable that do not vary across patient admissions. $\beta_{1}$ and $\beta_{2}$ are the coefficients of $X_{ij}$ and $Z_{j}$. $\beta_{0}$ is the adjusted average hospital intercept across all hospitals in the sample, and $\sigma_{u}^{2}$ is the between-hospital variance component.

We performed modeling based on a pooled data, and the parameters of the model were estimated separately for each disease. Specifically, we included both patient and hospital characteristics in our empirical models according to previous studies [24,34,35,36]. Patient characteristics included age, gender, admission source, admission status, insurance type, length of stay, and Elixhauser index. Admission source had two categories: Outpatient visit and emergency visit. Admission status indicated patients’ condition of disease on admission and were classified into three groups, namely, acute, urgent and general conditions. Hospital characteristics included hospital accreditation (tertiary vs. secondary), bed size, number of doctors per 100 beds, number of nurses per 100 beds, and hospital volume. We used expected hospital volume $N_{j}^{s}$ (increased per 100 cases) in our regression models because of the potential endogenous bias introduced by actual hospital volume [23]. The year trends were controlled using dummy variables. All data management and visualization were processed in RStudio (Version 1.1.442), while the statistical analyses were performed in Stata (Version 14.0, Stata Crop, Chicago, IL, USA).

## 3. Results

In this study, 46,959 patient admissions with AMI and 44,063 pneumonia admissions were identified. The process of patient selection is presented in Figure 1. The summary statistics of patient and hospital characteristics from 2015 to 2017 are described in Table 1. The overall in-hospital mortalities for AMI and pneumonia patients were around 2.5% and 1.7%, respectively. For AMI patients, about half were admitted to hospitals through emergency department, and most sought care in public hospitals (nearly 90 percent) and tertiary hospitals (over 80 percent). AMI patients were mainly the elderly and men. For pneumonia patients, over 80 percent chose public hospitals, but only about 60 percent attended tertiary hospitals. Compared to AMI patients, the percentage of admitted emergency room pneumonia patients was lower. Additionally, patients with pneumonia had a lower Elixhauser index and fewer secondary diagnoses.

**Figure 2:**
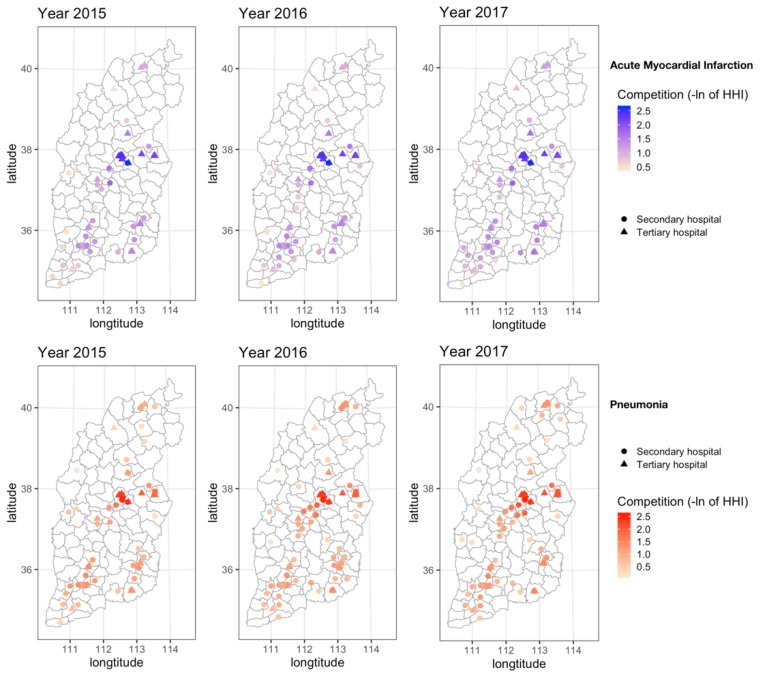
Geographical distribution of hospital competition for acute myocardial infarction and pneumonia in Shanxi, 2015–2017.

The geographical distributions of hospital competition among AMI and pneumonia diagnoses from 2015 to 2017 are shown in Figure 2. There was a wide range of hospital competition scores, from a less competitive market to a highly competitive market. As expected, the hospital competition was most intense in the provincial capital or major cities of Shanxi, which has the largest concentration of tertiary and large hospitals. However, in the remote areas of Western Shanxi where high-quality medical resources are scarce, the competition between hospitals was relatively weak. The average competition measures (−ln of HHI) for AMI were 1.55, 1.63 and 1.60 in 2015, 2016 and 2017, respectively, while the competition measures for pneumonia were 1.50, 1.66 and 1.53 in 2015, 2016 and 2017, respectively (Table 1).

Table 2 displays the estimated results of the relationship between hospital competition and in-hospital mortality for AMI and pneumonia separately. The intra-class correlation coefficients of the null models for AMI and pneumonia were 0.10 and 0.35, respectively, suggesting that a nest structure of data exists in our study. The odds ratios for the negative natural logarithm of HHI indicates that the hospital competition had a mixed effect on quality. Increasing hospital competition is correlated with higher risk of in-hospital death for pneumonia diagnosis (Odds Ratio (OR): 1.99; 95% Confidence Interval (CI): 1.51–2.64); the hospital competition reduces AMI quality, although the OR is insignificant (OR: 0.94, 95% CI: 0.77–1.11). The coefficient of competition for pneumonia suggested that increasing intensity of competition or decreasing hospital concentration was related to excess in-hospital mortality.

## 4. Discussion

In this study, we investigated hospital competition effects on in-hospital mortality, using the front pages of inpatients’ medical records of AMI and pneumonia cases from hospitals in Shanxi, China. Our results suggested that competition had a mixed effect on quality of care in AMI and pneumonia patients, and as such may differ by disease.

Many studies on competition and quality chose AMI patients as their representative population [20,22,23,27], and reported that competition could save lives. Consistent with the findings of Colla et al. [27], we found that increasing competition for AMI patients was associated with lower in-hospital mortality, but the effect was weak and insignificant. We inferred that the lack of evidence of the effect of competition on AMI quality could result from the low demand elasticity for AMI treatment. On one hand, there are limited hospital choices for AMI patients because of geographical distance, service capacity, operation conditions, and hospital reputation. On the other hand, since time to treatment is vital to the prognosis of AMI, these patients are usually admitted to the nearest hospital via emergency rooms, suggesting that they have less autonomy when choosing a hospital for care and exhibit lower levels of elasticity for demand in regards to hospital treatment [37]. As a result, hospitals have fewer incentives to compete on quality for AMI patients directly [20,23].

However, the effect of competition on the quality of pneumonia patient care showed a different pattern. Our findings are consistent with the study conducted by Gowrisankaran and Town [23], who concluded that competition reduced the quality of care for Medicare patients with pneumonia. In our study, increasing competition for pneumonia patients is associated with higher in-hospital mortality. There may be three explanations for this relationship. First, patients’ preferences in seeking care may only partially explain such results. Since there is no effective referral system in China, patients with severe conditions tend to bypass the primary hospitals and directly seek care in high-level hospitals, particularly in tertiary hospitals [4,9], which are usually concentrated in urban areas with competitive health markets. While we controlled for patients’ characteristics such as age, gender, admission status, and comorbidity index, patients with severe conditions naturally have a higher risk of mortality than other patients. Therefore, in-hospital mortality for those hospitals in a competitive market is more likely higher than other hospitals. Second, in China, patients have no official platform to get comprehensive, unbiased and detailed information of hospital operations and outcomes, even for public hospitals, especially regarding quality and safety of a specific hospital [5]. In the absence of reliable information about hospital service delivery, patients are attracted by technology and facilities (e.g., beds and medical equipment). Therefore, the limited public information allows hospitals to divert more resources to the medical arms race in market competition rather than quality improvement to attract more patients. Third, a large number of patients surging into the large public hospitals increases the medical staff’s workload [38], which has a potential negative effect on the quality of care [39,40].

Since most empirical evidence is based on data from developed countries, such as the US [20,23], the UK [21], the Netherlands [15], and Australia [24], it might not be appropriate to generalize these results to China, a developing country maintaining a unique tiered health care system with partially regulated prices. Pan et al. [6] discussed the competition effect on both outpatient and inpatient outcomes in China, using quality and cost indicators such as emergency mortality rate, waiting time, drug percentage in total cost, and total expenditure. They found that higher competition was associated with better outcomes for outpatient visits, while the effect of competition on quality and cost of inpatient visits was insignificant. In contrast, our study focused on inpatient services and used AMI and pneumonia patients as representative populations, and we found that the competition effects on quality of inpatients varied by their medical conditions. Furthermore, increasing competition reduced the quality of pneumonia patients, but had little effect on the quality of AMI patients. Compared with inpatients, outpatients have more autonomy in choosing a hospital to seek care and are more likely to make optimal choices based on their experience or hospitals’ reputations. As a result, hospitals under intense market competition have more incentives to build their reputation in outpatient services rather than inpatient services [6].

Compared with previous studies focusing on AMI and pneumonia [20,23], the overall in-hospital mortalities of these diseases in this study were relatively low (2.5% for AMI; 1.7% for pneumonia). Withdrawal from treatment is common in China due to many patients’ reluctance to die in hospitals [41]. Thus, the patients who leave hospitals and die at home are unable to be accounted for using in-hospital mortality, and thus observed in-hospital mortality is relatively low. This may bias our estimation of the competition effects. To address this issue, it is necessary to link the inpatient discharge records database in National Health and Family Planning Commission and the population death register information system managed by Ministry of Public Security through the patients’ unique IDs. However, it is common that the databases are administrated by different ministries, which hinders the linkage between different databases in China’s health care delivery system.

It is worth noting that patient insurance status had a significant effect on quality. Compared with NCMS patients, patients with URBMI and UEBMI had higher risk of in-hospital death. In our previous study focusing on patients admitted between 2014 and 2015 [42], however, NCMS patients with AMI had higher risk of mortality than UEBMI patients. This inconsistency of results may attribute to the integration of the URBMI and NCMS in China, launched in 2016 [43]. As the State Council of People’s Republic of China announced, the URBMI and NCMS would be unified in the following six areas: insurance coverage, insurance finance, payment standard, medicine and medical service item catalogue, management of the qualified insurance institutions, and insurance accounting [43]. According to this study, the patients with NCMS seemed to benefit from the reform of insurance integration in Shanxi. However, we have to emphasize that the insurance integration is a complex and long-term reform, and the effects of insurance on patient outcomes may not be clear during the transition period (2015–2017). The long-term effect of this reform on improving patient outcomes requires further exploration.

There are several limitations of our study. First, we only included two medical conditions in our analyses; the results of this study are difficult to generalize to other diseases or surgeries. Further studies focusing on other medical conditions, even full hospitalization, are warranted. Second, we incorporated only in-hospital mortality as the measure of quality in our study, which may not capture the comprehensive quality of hospital care. Moreover, the deaths occurred after withdrawal from treatment were not included in our study due to data unavailability, which may bias the estimations of hospital competition effects on quality. Future research using different indicators to measure quality of care in hospital, such as 30-day mortality, readmission, failure-to-rescue, and complication rates, may provide more comprehensive and robust evidence of the association between competition and quality. Third, the administrative data used in our study are limited in capturing potential confounders for patients’ mortality. Clinical information on diagnoses, tests, and treatments that may influence patient outcome were not included in our study due to unavailability; further research using more comprehensive data like clinical registries is recommended. Fourth, the economic capacity of patients may potentially influence patient choice and intensity of hospital competition, which may bias our results. However, the measures of economic capacity were difficult to control for this study due to a lack of data. Further research considering the effect of economic capacity of patients is necessary. Fifth, mutual referral system between low and high-tier hospitals may impact the competition pattern. Due to the deficiency of data, we did not test the robustness of our results by excluding the referral patients. Despite these limitations, our study had two main strengths. First, to the best of our knowledge, this is the first empirical study to investigate the effect of hospital competition on quality of care using the comprehensive administrative data from hospitals in China. It contributes to the limited evidence of the effect of hospital competition on quality in a developing country. Second, this study uses a measure of competition based on the predicted patients flow approach, including the potential endogenous factors (e.g., hospital quality and patient health status) influencing patient choice of hospitals.

## 5. Conclusions

Increasing competition for pneumonia patients appears to reduce quality, while increasing competition for AMI patients has negligible effect on hospital quality. Consequently, simply encouraging hospital competition may not provide effective channels to improve inpatient quality of health care in China’s current health care system.

## Figures and Tables

**Table 1:** Descriptive statistics of the patient and hospital characteristics, 2015–2017.

	Acute Myocardial Infarction			Pneumonia		
	2015	2016	2017	2015	2016	2017
**Main Interest**						
Competition (−ln of HHI)	1.55 ± 0.50	1.63 ± 0.46	1.60 ± 0.46	1.50 ± 0.67	1.66 ± 0.70	1.53 ± 0.64
**Outcome variable**						
Death, n (%)	393 (2.7)	471 (2.8)	346 (2.2)	197 (1.7)	325 (1.9)	215 (1.4)
**Patient characteristics**						
Age (year), mean ± SD	61.52 ± 12.66	61.84 ± 12.81	61.79 ± 12.48	65.21 ± 17.42	64.70 ± 18.14	65.14 ± 17.58
Female, n (%)	3736 (25.3)	4163 (24.9)	3727 (24.1)	4558 (40.1)	7282 (43.1)	6659 (42.1)
**Insurance**						
NCMS, n (%)	6593 (44.7)	7727 (46.2)	7425 (48.0)	4156 (36.6)	5698 (33.7)	5648 (35.7)
URBMI, n (%)	948 (6.4)	1227 (7.3)	993 (6.4)	850 (7.5)	1515 (9.0)	1437 (9.1)
UEBMI, n (%)	5114 (34.7)	5528 (33.1)	5084 (32.8)	4716 (41.5)	7098 (42.0)	6402 (40.5)
Self-payment, n (%)	1347 (9.1)	1364 (8.2)	1380 (8.9)	972 (8.6)	1393 (8.2)	1275 (8.1)
Other, n (%)	755 (5.1)	873 (5.2)	601 (3.9)	662 (5.8)	1192 (7.1)	1049 (6.6)
Emergency visit, n (%)	6851 (46.4)	7752 (46.4)	7565 (48.9)	1941 (17.1)	3096 (18.3)	2971 (18.8)
**Admission status**						
General, n (%)	8470 (57.4)	9035 (54.0)	8077 (52.2)	9996 (88.0)	14613 (86.5)	13612 (86.1)
Acute, n (%)	3360 (22.8)	4288 (25.6)	4220 (27.3)	1111 (9.8)	1804 (10.7)	1655 (10.5)
Urgent, n (%)	2927 (19.8)	3396 (20.3)	3186 (20.6)	249 (2.2)	479 (2.8)	544 (3.4)
**Severity**						
Low severity, n (%)	1549 (10.5)	1455 (8.7)	1074 (6.9)	3532 (31.1)	5047 (29.9)	4288 (27.1)
Moderate severity, n (%)	7353 (49.8)	7786 (46.6)	7035 (45.4)	5030 (44.3)	7116 (42.1)	6524 (41.3)
High severity, n (%)	5855 (39.7)	7478 (44.7)	7374 (47.6)	2794 (24.6)	4733 (28.0)	4999 (31.6)
Elixhauser index, mean ± SD	7.24 ± 5.96	7.85 ± 5.88	7.82 ± 5.89	3.97 ± 5.36	4.13 ± 5.44	4.32 ± 5.55
Length of stay (days), mean ± SD	12.01 ± 6.01	11.68 ± 5.91	11.19 ± 5.53	12.30 ± 7.41	12.26 ± 7.15	12.06 ± 7.08
**Hospital characteristics**						
Tertiary hospital, n (%)	12213 (82.8)	13853 (82.9)	12996 (83.9)	7046 (62.0)	9521 (56.4)	8614 (54.5)
Public hospital, n (%)	12999 (88.1)	14644 (87.6)	13416 (86.6)	8975 (79.0)	14472 (85.7)	13651 (86.3)
Bed size, mean ± SD	1023.58 ± 472.29	1048.01 ± 497.81	1115.27 ± 490.16	953.00 ± 547.65	930.22 ± 578.98	919.77 ± 563.66
Number of doctors per 100 beds, mean ± SD	39.22 ± 7.74	39.88 ± 8.61	40.30 ± 8.44	38.64 ± 7.91	40.65 ± 10.45	40.60 ± 9.53
Number of nurses per 100 beds, mean ± SD	59.53 ± 15.73	63.66 ± 13.14	64.78 ± 13.65	55.59 ± 18.29	61.89 ± 18.51	61.02 ± 17.72
Expected volume, mean ± SD	382.54 ± 151.26	436.69 ± 171.86	405.15 ± 162.85	246.84 ± 119.36	290.30 ± 136.90	260.15 ± 115.86
Total, n (%)	14,757 (31.4)	16,719 (35.6)	15,483 (33.0)	11,356 (25.8)	16,896 (38.3)	15,811 (35.9)

Note: HHI, Herfindahl-Hirschman Index; NCMS, the rural new cooperative medical scheme; SD, standard deviation; UEBMI, the urban employee-based basic medical insurance; URBMI, the urban resident-based basic medical insurance scheme.

**Table 2:** Random-intercept logistic regression: estimates of hospital competition effect on quality.

	Acute Myocardial Infarction		Pneumonia	
	OR	95% CI	OR	95% CI
Competition (−ln of HHI)	0.94	(0.77–1.11)	1.99	(1.51–2.64)
Age	1.06	(1.05–1.07)	1.05	(1.04–1.06)
Gender				
Male	Ref.		Ref.	
Female	1.38	(1.21–1.57)	0.78	(0.65–0.93)
Admission source				
Outpatient visit	Ref.		Ref.	
Emergency visit	1.56	(1.35–1.79)	1.45	(1.20–1.73)
Admission status				
General	Ref.		Ref.	
Acute	0.96	(0.80–1.16)	1.66	(1.33–2.06)
Urgent	2.76	(2.36–3.24)	6.86	(5.48–8.59)
Insurance				
NCMS	Ref.		Ref.	
URBMI	1.62	(1.30–2.01)	2.26	(1.53–3.32)
UEBMI	1.78	(1.53–2.07)	2.94	(2.18–3.96)
Self-payment	0.98	(0.74–1.30)	1.79	(1.18–2.73)
Others	1.44	(1.04–2.01)	3.33	(2.30–4.82)
Elixhauser index	1.03	(1.02–1.04)	1.09	(1.08–1.10)
Length of stay	0.87	(0.67–0.97)	1.00	(0.99–1.01)
Hospital grade				
Secondary hospital	Ref.		Ref.	
Tertiary hospital	1.38	(0.77–2.47)	1.55	(0.92–2.64)
Ownership				
Private hospital	Ref.		Ref.	
Public hospital	1.73	(1.12–2.67)	1.91	(1.24–2.95)
Beds	0.95	(0.92–0.98)	0.99	(0.95–1.04)
Number of doctors per 100 beds	1.00	(0.99–1.01)	1.01	(0.99–1.03)
Number of nurses per 100 beds	1.00	(0.99–1.01)	1.01	(0.99–1.02)
Expected volume (per 100 cases)	1.01	(0.99–1.02)	1.00	(0.99–1.01)
Year				
2015	Ref.		Ref.	
2016	0.97	(0.73–1.30)	0.78	(0.54–1.14)
2017	0.84	(0.63–1.13)	0.54	(0.37–0.78)

Note: OR, odds ratio; CI, confidence interval; HHI, Herfindahl-Hirschman Index; NCMS, the rural new cooperative medical scheme; UEBMI, the urban employee-based basic medical insurance; URBMI, the urban resident-based basic medical insurance scheme.

## Abbreviations

The following abbreviations are used in this manuscript:

AMI	Acute Myocardial Infarction
CI	Confidence Interval
ECI	Elixhauser Comorbidity Index
HHI	Herfindahl-Hirschman Index
ICD-9	The International Classification of Disease, Ninth Revision
ICD-10	The International Classification of Disease, Tenth Revision
NCMS	The Rural New Cooperative Medical Scheme
OR	Odds Ratio
UEBMI	The Urban Employee-based Basic Medical Insurance Scheme
URBMI	The Urban Resident-based Basic Medical Insurance Scheme
